# Bee on Boron—Sufficient Boron Supply of *Brassica napus* Is Crucial for Attracting Pollinating Insects to Ensure Seed Yield

**DOI:** 10.1002/ece3.72895

**Published:** 2026-01-12

**Authors:** Jiline B. Tölle, Paula Prucker, Johanna Saumweber, Thomas D. Alcock, Sara D. Leonhardt, Gerd Patrick Bienert

**Affiliations:** ^1^ Crop Physiology, TUM School of Life Sciences Technical University of Munich Freising Germany; ^2^ HEF World Agricultural Systems Center Technical University of Munich Freising Germany; ^3^ Plant‐Insect Interactions, TUM School of Life Sciences Technical University of Munich Freising Germany

**Keywords:** boron deficiency, inflorescence, insect decline, pollinators, rapeseed, seed yield

## Abstract

A sufficient supply of the micronutrient boron (B) is crucial for plant development and fertility. Boron limitations in crops, such as rapeseed ( *Brassica napus* ), cause deformed or infertile flowers and may consequently affect yield both directly and indirectly via reduced flower fertility or insect pollination, respectively. This latter point is particularly relevant for rapeseed, as its yield increases via insect pollination, which relies on healthy‐looking flowers to attract pollinators. This study aimed to quantify the direct and indirect effects induced by B limitation on yield. Thus, we determined the extent to which B‐deficiency in rapeseed affects plant–pollinator interactions by cultivating B‐inefficient (*Daktari* and *CR3153*) and B‐efficient (*CR2267*) cultivars at B‐sufficient and B‐deficient substrate levels in pot trials and exposing them to a natural pollinator community in a cafeteria experiment. All cultivars displayed phenotypic B‐deficiency symptoms at B‐deficient levels with 9% of the total flowers appearing deformed in B‐efficient *CR2267* compared to 34% and 18% in *Daktari* and *CR3153*, respectively. Both B‐inefficient cultivars were visited by a higher number of pollinating insects at B‐sufficient levels (*Daktari* by 59%, *p* < 0.001; and *CR3153* by 37%, *p* = 0.002) than under B‐deficiency. Compared to B‐inefficient cultivars, pollinator visits to *CR2267* were less abundant and not affected by B‐deficiency (*p* = 0.252). Across cultivars, pollinator visitation decreased with an increase in the number of deformed flowers (*p* < 0.001). Insect pollination increased seed yield compared exclusively to self‐pollination only for the B‐inefficient rapeseed cultivars by around 20% on either B‐deficient or B‐sufficient supply levels (*p* < 0.05), but not for *CR2267*, suggesting a cultivar‐dependent effect of insect pollination on yield. Sufficient B supply is crucial for successful rapeseed flower growth and fertility as well as for attracting pollinating insects, thus securing yield in a pollinator‐dependent crop.

## Graphical abstract

The study demonstrates that sufficient B supply is crucial for successful rapeseed flower growth and fertility as well as for attracting pollinating insects, thus securing yield in a pollinator‐dependent crop. It is illustrated that climate change can pose a threat to crop yield via both direct effects, that is, an impaired nutrient uptake, and indirect effects, that is, reduced pollinator visitation.

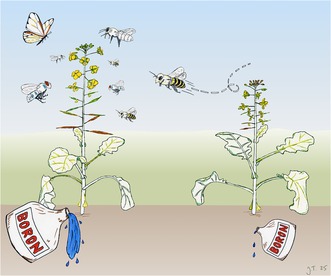

## 1 Introduction

Boron (B) is known as an essential micronutrient for vascular plants for more than 100 years (Warrington 1923; Wimmer et al. 2020). Boron is crucial for plant growth, development, and fertility. A sufficient B supply is therefore particularly important for the yield of crops with a high B demand, such as fruit trees (e.g., apples and cherries) or species of the *Brassicaceae* family, including the economically important crop rapeseed ( *Brassica napus* L.) (Cakmak et al. 2023; Shorrocks 1997). Despite this knowledge, B‐deficiency has been observed in more than 80 countries and represents a globally occurring problem in agricultural ecosystems, especially in countries with a high production of rapeseed, such as Canada and China, as well as throughout Europe (FAO 2025; Shorrocks 1997). The reasons for reduced plant B availability are manifold: In soils with pH levels above 7 or with high organic matter, B adsorption to soil particles occurs as borate anions. In the form of undissociated boric acid molecules, which are quantitatively the major plant‐available chemical B species in most agricultural soils, B is highly soluble and therefore easily leached during rainfall (Cakmak et al. 2023; Shorrocks 1997). On the other hand, limitations of soil water, for example, during drought periods, reduce the mass flow of essential plant nutrients in general, but especially of highly soluble nutrients such as B, consequently decreasing their delivery to plant roots, the location of nutrient uptake (Barber 1995; Wimmer and Eichert 2013). Drought periods in Central Europe have intensified in spring and summer in recent years, and climate predictions forecast that such weather situations will occur more frequently in the future (Grillakis 2019; Marx 2024). These droughts coincided with the flowering time of rapeseed, a developmental stage that is highly prone to symptoms of B‐deficiency. Consequently, losses in crop production have been observed and have been partly explained by B‐deficiency induced effects.

The molecular role of B in plants is to cross‐link two rhamnogalacturonan‐II monomers in the pectin fraction of primary cell walls in order to physiologically regulate their stability and flexibility (O'Neill et al. 1996). Thus, B‐deficiency negatively affects all plant organs and tissues, particularly flower development and fertility, due to the rapid growth rates of reproductive tissues depending on the controlled formation of cell walls with defined properties. Besides wrinkled/non‐turgid petals, B‐deficiency leads to impairments in pollen grain development and germination, in pollen tube growth and cell wall thickening of the stigma, resulting in sterile flowers (Dell and Huang 1997; Zhang et al. 2017). Accordingly, frequently occurring typical B‐deficiency symptoms in rapeseed inflorescences are either deformed flowers or healthy‐appearing flowers, which do not produce seeds (i.e., “flowering without seed setting” phenotype) (Tölle et al. 2025; Verwaaijen et al. 2023; Zhang et al. 2014). Such B‐deficient flowers form short, thin, undeveloped siliques with no or undersized and shriveled seeds, causing a significant yield reduction. Around 33% and 4.6% of the worldwide oilseed rape production was harvested in Europe and Germany in 2023, respectively. Moreover, Europe and Germany outperformed the worldwide average oilseed yield of 2.1 t ha^−1^ in 2023 by 36% and 69%, respectively (FAO 2025). Rapeseed was with 43 million (M) ha, the second most harvested oilseed crop after soybean (137 M ha) and the sixth most harvested crop after wheat, maize, rice, soybean, and barley worldwide in 2023 (FAO 2025), demonstrating its significance as a crop for food, feed, and fuel production. A total of 35% of the global food production and more than 75% of the world's food crops are entomophilous and depend on pollinators for fruit set and yield (Klein et al. 2007; Turo et al. 2024). Insect pollination also significantly enhances yield parameters in *Brassicaceae* species, such as the self‐incompatible turnip rape ( *Brassica rapa* ) and cabbage ( *Brassica oleracea* ) species (Badenes‐Pérez 2022). In the self‐compatible rapeseed, being an allopolyploid hybrid resulting from a hybridization of *B. rapa* and *B. oleracea* , pollination generally occurs through both self‐pollination and cross‐pollination. The latter is typically achieved by a combination of wind and insect pollination (Bommarco et al. 2012; Garratt et al. 2014). While self‐pollination is sufficient to produce seeds, insect pollination often increases fruit and seed set in self‐compatible species (Badenes‐Pérez 2022; Williams et al. 1986; Zou et al. 2017). Seed set depends on the number of pollen grains reaching the stigma (Pechan 1988; Zou et al. 2017), which is increased by pollinators (Abrol 2007; Zou et al. 2017). The main pollinators of rapeseed are bees (Apoidae—honey bees [ *Apis mellifera* ], bumble bees [*Bombus* spp.], mining bees [*Andrenidae* spp.], sweat bees [*Halictidae* spp.], and other wild bees), and hoverflies (Syrphidae) (Abrol 2007; Badenes‐Pérez 2022; Bommarco et al. 2012; Garratt et al. 2014; Westphal et al. 2003). These pollinators are attracted by floral rewards, such as nectar and pollen, and floral cues, such as flower shape, scent, and color (Cook et al. 2005; Lawson and Rands 2018; Leonhardt et al. 2024), with the bright yellow rapeseed flowers strongly attracting pollinators from a distance (Haneklaus et al. 2005; Kazda et al. 2019).

In well‐fertilized rapeseed, insect pollination increases seed yield by up to 40% compared to self‐pollination (Garratt et al. 2018). No positive effect of insect pollination was found on reduced macronutrient fertilizer treatments (Garratt et al. 2018). Several studies investigated the effects of nitrogen (N), nitrogen–phosphorus–potassium (NPK), or sulfur (S) fertilizers on flowers and floral rewards (Burkle and Irwin 2010; Dupont et al. 2018; Garratt et al. 2018; Haneklaus et al. 2005; Russo et al. 2023; Sengupta and Krishna 2023). Sulfur deficiency in rapeseed flowers results in significant morphological changes in size and shape, and a change of the petal color from bright to pale yellow or even white. Consequently, S‐deficiency causes a differential attractiveness of S‐deficient and sufficiently S‐supplied rapeseed plants for honey bees (Haneklaus et al. 2005). In contrast to studies investigating the effects of macronutrients on plant–pollinator interactions, little is known about such effects of micronutrients and especially those known to impact flower physiology, such as B. Besides ensuring a sufficient nutrient supply via fertilization, the cultivation of nutrient‐deficiency‐tolerant cultivars represents an alternative strategy to achieve stable yields even under suboptimal growth conditions. In rapeseed, different B‐deficiency tolerance mechanisms were suggested and identified, either involving a more effective root B uptake or B translocation from roots to shoots (Hua et al. 2016; Liu et al. 2024; Zhang et al. 2017), or a more efficient utilization of B within the plant (Pommerrenig et al. 2018). Whether B‐deficiency tolerance mechanisms in rapeseed affect plant–insect interactions and pollination has not yet been investigated.

In this study, we hypothesized that reduced plant B availability, which leads to deformed inflorescences and potentially floral rewards, negatively impacts (i) pollinator visits and (ii) subsequently yield. Therefore, we investigated the abundance and richness of insect groups visiting rapeseed plants cultivated on B‐sufficient and B‐deficient conditions, and whether insect pollination increased yield under B‐deficient conditions. Defined B availabilities were adjusted in pot trials using a soil substrate cultivation system (Tölle et al. 2025), allowing the induction of B‐deficiency symptoms precisely during flowering in plants that had developed normally until then. Potential differences in insect visits and plant yield were assessed in three rapeseed cultivars differing in their B‐efficiency: B‐efficient: *CR2267* (fodder‐type), B‐inefficient: *CR3153* (oilseed‐type), and *Daktari* (oilseed‐type).

## 2 Materials and Methods

### 2.1 Plant Material

Three rapeseed ( *Brassica napus* ) cultivars were used for this study: B‐efficient *CR2267* (cultivar “Hiyauchina,” fodder type, semi type), B‐inefficient *CR3153* (cultivar “Tower,” oilseed type, spring type) (Pommerrenig et al. 2018), and *Daktari* (hybrid, oilseed type, winter type; Breeder Rapool), a currently cultivated German high‐seed‐yield cultivar.

### 2.2 Substrate Preparation and Plant Cultivation

During flowering, rapeseed plants were cultivated on B‐sufficient (2.5 mg B kg^−1^ substrate) and B‐deficient (0.4 mg B kg^−1^ substrate) substrate conditions for pollinator observations.

Plants were sown directly in pots (Ø 13 cm) in a glasshouse trial at the TUM Plant Technology Center in Dürnast (48°24′17″ N, 11°41′38″ E). Pots were filled with 350 g (±10 g) of a white‐peat volcanic clay mixture, which is a B‐free soil‐substrate (“Fruhstorfer Nullerde,” < 0.08 mg B kg^−1^ substrate) (Eggert and von Wirén 2017). To adjust the pH, the substrate was supplemented with 0.3% CaCO_3_ and 0.2% CaO by mixing 20 kg substrate batches in a clean cement mixer (Altrad Lescha GmbH, Typ SBM P 150L, Burgau, Germany) for 10 min. Afterwards, several batches were mixed and dried for 2 weeks (dry matter ≈55%).

Nutrient solutions were prepared by mixing stock solution with ultrapure water (18.2 MΩ; Merck, Milli‐Q IQ‐7000 Ultrapure Water System, Darmstadt, Germany) in 1 L plastic bottles (Nalgene, Nalge Nunc International Inc., Rochester, NY, USA): 100× macro‐element mix (60 g L^−1^ NH_4_NO_3_, 40 g L^−1^ KH_2_PO_4_, 20 g L^−1^ MgSO_4_, and 6 g L^−1^ K_2_SO_4_), 100× micro‐element mix (4 g L^−1^ MnCl_2_ × 4H_2_O, 1.3 g L^−1^ CuSO_4_ × 5H_2_O, 1.3 g L^−1^ ZnSO_4_ × 7H_2_O, 8.5 mg L^−1^ NaMoO_4_ × 2H_2_O) and 100× iron mix (0.7 g L^−1^ NaFeEDTA). All nutrient solutions were applied once at sowing and from BBCH50 (inflorescence emergence) onwards every second week, containing the following nutrients: 15 mM NH_4_NO_3_, 5.9 mM KH_2_PO_4_, 1.6 mM MgSO_4_, 780 μM K_2_SO_4_, 404 μM MnCl_2_, 104 μM CuSO_4_, 90 μM ZnSO_4_, 40 μM NaFeEDTA, and 0.7 μM NaMoO_4_. A 100× boric acid stock (1.4 g L^−1^ H_3_BO_3_) was prepared in a separate glass bottle. With this exception, no glassware was used during the experiment to prevent contamination with additional B.

Boron efficiency of *Daktari* was rated by comparing the biomass growth at the B‐deficient (< 0.08 mg B kg^−1^ substrate) and B‐sufficient (2.5 mg B kg^−1^ substrate) levels at 28 days after sowing (DAS).

Plants for pollinator observation were cultivated at a B‐sufficient level of 2.5 mg B kg^−1^ substrate (*2.5B*; 226 μM H_3_BO_3_) and initially at a B‐deficient level of 0.25 mg B kg^−1^ substrate (*0.25B*; 22.6 μM H_3_BO_3_). For each cultivar and B level, 60 plants (*n* = 60) were cultivated in the glasshouse with one plant per pot. On *0.25B*, all plants of *CR2267* and *CR3153* and 40% of the plants of *Daktari* were given an additional B application of 13.6 μM H_3_BO_3_ at BBCH53 to reach a B‐deficient level of 0.4 mg B kg^−1^ substrate (*0.4B*; 36.2 μM H_3_BO_3_). Sixty percent of the B‐deficient plants of *Daktari* were kept at the original B‐deficient concentration of *0.25B* because their development had already progressed to a stage (first flowers open, BBCH60) where additional B supply would have significantly mitigated the B‐deficiency symptoms. While the *2.5B* condition ensured sufficient B supply during the entire plant development, the B‐deficient conditions were titrated so that visible B‐deficiency symptoms were induced not earlier than in the inflorescence at the onset of flowering.

Rapeseed developmental stages were tracked against the “Biologische Bundesanstalt, Bundessortenamt, und Chemische Industrie” (BBCH) growth scale (Lancashire et al. 1991).

Plants were cultivated in the glasshouse under conditions that ensured flowering in spring for pollinator observation (Table S1 for climate data). *Daktari* was sown in November (17.11.2022), and *CR2267* and *CR3153* were sown in January (10.01.2023). At BBCH15, plants were vernalized for several weeks (5°C, 8 h day/16 h night, 180–200 μmol photons PAR m^−2^ s^−1^ light intensity) so that they flowered close to each other in spring 2023 for pollinator observation. To avoid subsequent B contamination, plants were placed only together within each B level on a watering tray with six plants per tray. Plants were well‐watered with ultrapure and B‐free deionized water depending on the substrate moisture and pot weight.

### 2.3 Inflorescence Phenotype

Several inflorescence parameters were determined during flowering to measure the severity of the B‐deficiency symptoms and to determine their effect on plant–pollinator interactions. At BBCH60, the date of the start of flowering (i.e., when the first flower bud on the main raceme had opened with visible yellow petals) was recorded for each plant. At least half of the plants of each cultivar at each B substrate level (*n* = 30, for Daktari on *0.4B*, *n* = 22) were phenotyped during flowering at 4 DAF (days after flowering started), when the first B‐deficiency symptoms appeared. Plants were phenotyped again at 14 DAF when the main raceme had, on average, fewer than 10 closed flower buds left on B‐sufficient conditions. The following parameters of the main raceme were determined: the number of flowers with turgid petals and without B‐deficiency symptoms (in this study, referred to as “asymptomatic flowers”) and the number of flowers displaying B‐deficiency symptoms such as non‐turgid/wrinkled petals, extended stigma, and/or anther‐stigma separation (in this study, referred to as “deformed flowers”). At 14 DAF, the number of side racemes with asymptomatic and/or deformed flowers was counted. The main shoot length was determined during fruit development (BBCH70) when no further shoot elongation occurred.

### 2.4 Measuring Tissue B Concentrations via ICP‐MS Analysis

The B concentration in the inflorescence (i.e., flowers, flower buds, shoot apical meristem, and pedicels) of the upper 6–8 side racemes (pooled per plant) of four randomly chosen plants per cultivar and B level was measured 3 weeks after flowering had started. All samples were dried in a forced‐air oven at 50°C, and the dry weight (DW) was recorded after reaching weight constancy. The sample material was then ground to a fine powder using a ball mill (Retsch GmbH, Typ MM301, Haan, Germany).

For elemental analysis, tissue subsamples (ca. 100 mg DW with actual weights recorded) of ground plant tissue samples were digested in 20 mL MARSXpress PFA digestion vessels (CEM GmbH, Kamp‐Lintfort, Germany) within a 40‐place turntable in a CEM MARS 6 microwave digestion system. The subsample material was digested in 2 mL 67% trace analysis grade nitric acid (HNO_3_; VWR NORMATOM, VWR International GmbH), 1 mL Milli‐Q water (18.2 MΩ.cm; Merck KGaA, Darmstadt, Germany), and 1 mL 30% trace analysis grade H_2_O_2_ (Suprapur, Merck—Supelco, Merck KGaA, Darmstadt, Germany). The microwave was set to ramp up to 65°C over 5 min with a power of up to 100 W, at which temperatures were held for a further 5 min. Sample temperatures were then ramped up to 190°C over 15 min with a power of up to 900 W, and at which temperatures were held for 40 min. Two operational blanks were included in each digestion run, along with duplicate samples of the certified reference material (CRM) of leaf (Tomato SRM 1573a, NIST, Gaithersburg, USA). Following digestion, each tube was made up to a final volume of 14 mL by adding 10 mL Milli‐Q water, transferred to a 15 mL centrifuge tube (Cellstar, Greiner Bio‐One GmbH, Frickenhausen, Germany), and stored at room temperature. Tissue digestates were diluted 1‐in‐5 using Milli‐Q water prior to elemental analysis. The concentrations of the element B were obtained using inductively coupled plasma‐mass spectrometry (ICP‐MS; PerkinElmer NexION 350D, PerkinElmer, Rodgau, Germany). Boron was measured in the standard mode in which the reaction cell was evacuated. Samples were introduced using an Elemental Scientific 2DX autosampler (Elemental Scientific) through a MEINHARD concentric glass nebulizer (PerkinElmer) and into a NexION glass spray chamber (PerkinElmer). Scandium was used as an internal standard and was introduced via a separate line at a concentration of 10 μg L^−1^, in 2.5% trace analysis grade HNO_3_. A multielement standard solution (Multielement‐Standardlösung “HR‐ICP‐MS,” Bernd Kraft, Duisburg, Germany) was used to calibrate B in the range of 0.5–50 μg L^−1^. ICP‐MS operation and sample processing were managed using Syngistix software (PerkinElmer). The obtained concentration values were blank and weight‐normalized, and are reported here in mg kg DW^−1^. Average B recovery from CRM samples was 81% of certified values. The limit of detection (LOD) for B was calculated as three times the standard deviation of the operational blank concentrations, using a notional starting weight of 100 mg. Boron concentrations measured for all samples were above the LOD.

### 2.5 Pollinator Observations

Pollinator observations (“pollination treatment”) were divided into insect‐ and self‐pollination treatments and were conducted within the first 2 weeks of flowering of each cultivar in April and May 2023 at three locations around the glasshouse (approx. 75 m straight line distance between locations) with equal number of plants per cultivar and B level (*n* = 20) per location, including the *Daktari* plants on *0.25B*. As soon as at least two‐thirds of the plants per cultivar were flowering, the plants were taken out of the glasshouse in the morning and observed for visitations by potential pollinators for 1 h (“insect” treatment). To exclude pollination by insects and thus measure the degree of self‐pollination, the main raceme of one‐third of the plants at each B level and cultivar was covered with air‐permeable plastic bags (Crispac‐Beutel) at the beginning of the flowering period (“self” treatment). Besides the 1 h of observation time, plants were left outside for a minimum of 6 h to allow for further insect pollination and were brought back into the glasshouse in the late afternoon. Observations took place on days without precipitation, an outside temperature above 5°C, and a wind intensity below 20 km h^−1^ (for *CR2267*, below 15 km h^−1^). In total (whole observation period), *Daktari* was observed on 9 days, *CR3153* on 8 days, and *CR2267* on 6 days, for each 1 h, within the first 2 weeks of respective flowering. Afterwards, the plants remained in the glasshouse until harvest.

During the observations, every insect landing on a flower was caught with a *Drosophila* glass and stored in a cooling box. Each vessel was labeled with the respective cultivar and B level. Insects were caught from the flowers of the main raceme and side racemes. After 1 h of observation, all insects were individually checked and assigned to one of the following seven functional groups: beetles (all Coleoptera), butterflies (all Lepidoptera), flies (all Diptera excluding Syrphidae), hoverflies (all Syrphidae), bumble bees (all *Bombus* spp.), honey bees ( *Apis mellifera* ), and wild bees (all Apidae, excluding *Apis mellifera* and *Bombus* species). If the insect caught did not fit into one of the aforementioned groups, it was assigned to a separate group of “other insects” (e.g., bugs [Heteroptera] or wasps [e.g., Vespidae], which included overall 36 insects that were not included in any further analyses). The common pollen beetle ( *Brassicogethes aeneus* Fabricius) and very small, swarming individuals of a *Hymenoptera* species were also not counted.

### 2.6 Harvest

During the ripening stage (BBCH80‐89), beginning of June, watering was stopped to accelerate silique ripening. Harvest of the siliques of the previously phenotyped plants took place on 21st of June (*Daktari* and *CR3153*) and 28th of June (*CR2267*) 2023 (60–70 days after flowering started). For the plants of the “insect” and “self” treatment, only siliques of the main racemes that had developed during the period of possible insect pollination were harvested and counted. Siliques were categorized as (i) (well‐developed) siliques containing seeds, or (ii) short, thin, undeveloped siliques without any seeds. After drying the (well‐developed) siliques in a forced‐air oven at 50°C (until reaching weight constancy), seeds were harvested. Only large (Ø > 1 mm), round, brown to black seeds were further analyzed, whereas small (Ø < 1 mm), deformed seeds were excluded from the analysis. Seeds were re‐dried at 50°C to determine the dry weight after reaching weight constancy. Seeds were counted using a seed counter (Pfeuffer GmbH, Typ Contador, Kitzingen, Germany) equipped with a hopper for rapeseed.

### 2.7 Statistical Analysis

Statistics were conducted in MS Excel 2019 (Microsoft Corporation, Redmond, WA, USA), RStudio (R Core Team 2023; RStudio, PBC, Boston, MA, USA), and OriginPro (OriginPro 2021b [64‐bit] SR2; OriginLab Corporation, Northampton, MA, USA). Graphs were plotted in OriginPro and RStudio.

The effects of the cultivars and B levels on different phenotyping parameters and tissue B concentration were tested using two‐way analyses of variance (ANOVA), followed by post hoc Tukey's tests (*p* < 0.05).

Differences in the insect abundance per hour and insect group richness between cultivars and B levels, determined by observing 40 individual plants per cultivar and B level, were analyzed with a generalized linear mixed effect model (GLMM) using template model builder (Brooks et al. 2017) and Poisson distribution. Further detailed analyses with GLMMs were conducted only for those insect groups that comprised more than 10% of the total insects caught, that is, honey bees, flies, and wild bees (Table S3). Boron levels and cultivars were set as fixed effects in the GLMMs, while the number of trays and temperature were set as random effects, as both showed significant effects in preliminary correlation tests. Preliminary data testing revealed no significant effect of location, and hence, was not included as a random factor in the models. An emmeans (Lenth 2023) test for pairwise post hoc comparisons with Tukey's correction was used to subsequently test for differences between individual groups (*p* < 0.05).

Additionally, a Pearson's chi‐squared test of independence was used to test whether the total abundance of all visiting insects, as well as honey bee, wild bee, and fly abundances, depended on the B level for each cultivar individually (*p* < 0.05).

Correlation of deformed against asymptomatic flowers was tested using a Spearman correlation analysis. Correlations between total insect abundance and honey bee, wild bee, and fly abundance per hour and the number of asymptomatic flowers, as well as potential interactions with cultivar and B level, were investigated using a GLMM with number of trays and temperature as random effects and the number of asymptomatic flowers as a covariate. In addition, correlations between ratios of the results per *0.4B* versus *2.5B* (raw data on *0.4B* [and for *Daktari* additionally on *0.25B*] divided by the mean on *2.5B*) for total insect abundance, honey bee, wild bee, and fly abundance per hour, and the number of deformed flowers were analyzed using Spearman correlation.

To assess yield, the average number of seeds per silique (=number of seeds per silique/number of siliques) and the relative seed weight (=weight of seeds/number of seeds) were calculated. Differences in these parameters between the B level and pollination treatment were analyzed for each cultivar (*Daktari*, *CR3153*, and *CR2267*) using a linear mixed effects model (nlme package; Pinheiro et al. 2021). B levels and pollination treatment were set as fixed effects, while the tray number was set as a random effect. An emmeans (Lenth 2023) test for pairwise post hoc comparisons with Tukey's correction was used to test for differences between individual groups (*p* < 0.05).

In graphs with box plots, the boxes represent the 25th and 75th percentiles, with the median shown as a horizontal line and a small square as the mean. Whiskers represent the 1.5 × interquartile range, with any outliers shown. Error bars in bar charts represent the standard error.

## 3 Results

### 3.1 Boron‐Deficiency Reduces Plant Vigor and Resulted in Macroscopic Symptoms of B Deficiency in Inflorescences, With the B‐Efficient Cultivar Being Less Affected Than the B‐Inefficient Cultivars

**FIGURE 1:**
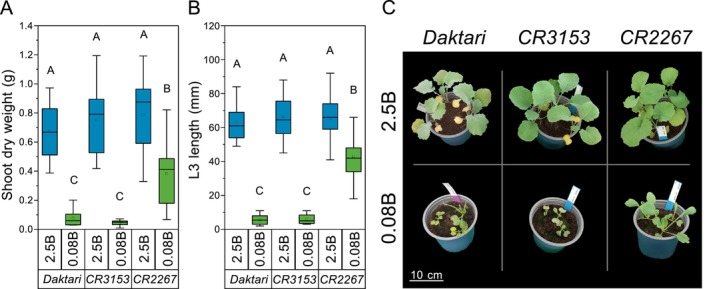
Boron (B) level and *Brassica napus* cultivar‐dependent shoot growth parameters and visual appearance. (A) Shoot dry weight, (B) length of leaf 3 (L3), and (C) shoot images of the rapeseed cultivars *Daktari*, *CR3153*, and *CR2267* at B‐sufficient (2.5 mg B kg^−1^ substrate; *2.5B*) and B‐deficient (0.08 mg B kg^−1^ substrate; *0.08B*) levels. (A, B) 28 days after sowing (DAS); (C) 21 DAS. Upper‐case letters indicate significant differences between cultivars and B levels (*p* < 0.05, two‐way ANOVA, post hoc Tukey's test) (*n* = 4–17).

While the rapeseed cultivars *CR2267* and *CR3153* were previously characterized as B‐efficient and B‐inefficient, respectively (Pommerrenig et al. 2018; Tölle et al. 2025), the cultivar *Daktari* was classified as rather B‐inefficient, as shoot dry weight (DW) and leaf 3 (L3) length were significantly (both *p* < 0.001) reduced by 89% and 86% on average, respectively, under B‐deficient (*0.08B*, 0.08 mg B kg^−1^ substrate) compared to B‐sufficient (*2.5B*, 2.5 mg B kg^−1^ substrate) growth conditions, similarly to *CR3153* (Figure 1A,B, Table S2A). *Daktari* developed visible B‐deficiency symptoms in the shoot, such as reduced biomass gain and lanceolate‐like deformed leaves (Figure 1C).

**FIGURE 2:**
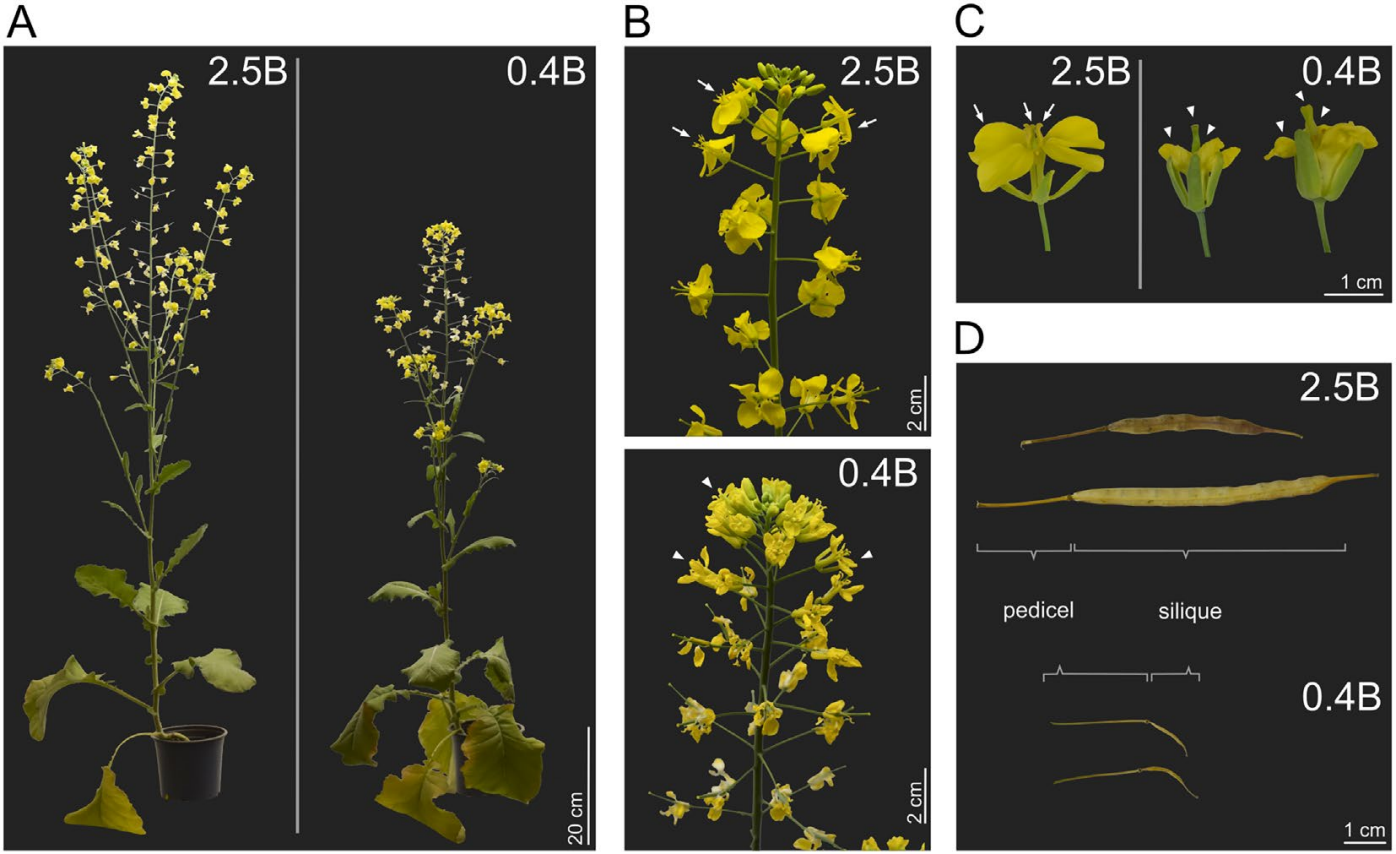
Images of *Brassica napus* shoot and inflorescence tissues grown on different boron (B) levels. Phenotypic effects of rapeseed plants grown at B‐sufficient (2.5 mg B kg^−1^ substrate; *2.5B*) and B‐deficient (0.4 mg B kg^−1^ substrate; *0.4B*) levels at 7 days after flowering (DAF): (A) whole plant, (B) inflorescence of the main raceme, and (C) individual asymptomatic (turgid petals, anther‐stigma proximity; indicated with arrows) (*2.5B*) and deformed (wrinkled petals, anther‐stigma separation; indicated with arrow heads) (*0.4B*) flowers, and (D) developed (*2.5B*) and undeveloped (*0.4B*) siliques.

To evaluate the effect of reduced B nutrition on the plants' interactions with pollinators, B‐deficient conditions were titrated at 0.4 mg B kg^−1^ substrate (*0.4B*), a B‐concentration at which B‐deficiency symptoms in rapeseed only became visible after the onset of flowering and only in the inflorescence. At *0.4B*, plants developed deformed flowers (wrinkled/non‐turgid petals, extended stigma, anther‐stigma separation) (Figure 2A–C) and the “flowering‐without‐seed‐setting” phenotype with seedless siliques at harvest (BBCH89, stage of ripe seeds) appeared (Figure 2D).

**FIGURE 3:**
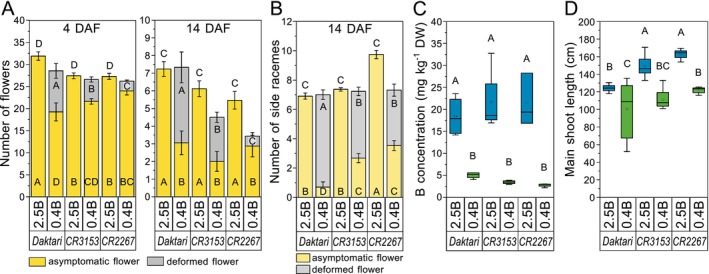
Boron (B)‐level and *Brassica napus* cultivar‐dependent flower and shoot parameters. (A) Number of asymptomatic and deformed flowers of the main raceme at 4 and 14 days after flowering (DAF), (B) number of side racemes with asymptomatic and deformed flowers at 14 DAF, (C) B concentration per kilogram dry weight (DW) of side racemes 3 weeks after flowering started, and (D) main shoot length at harvest of cultivars *Daktari*, *CR3153* and *CR2267* at B‐sufficient (2.5 mg B kg^−1^ substrate; *2.5B*) and B‐deficient (0.4 mg B kg^−1^ substrate; *0.4B*) levels. Upper‐case letters indicate significant differences between cultivars and B levels (*p* < 0.05, two‐way ANOVA, post hoc Tukey's test) (for [A, B]: *n* = 14–60; [C]: *n* = 3–4; and [D]: *n* = 10–13). (A, B) Error bars: ±SE.

During the inflorescence period, at 4 and 14 days after flowering started (DAF), plants grown on *2.5B* developed only asymptomatic flowers with turgid petals and no B‐deficiency symptoms on the main raceme and the side racemes (Figures 2 and 3A,B). At 4 DAF, on *0.4B* the B‐inefficient cultivars *Daktari* and *CR3153* developed significantly fewer asymptomatic flowers (on average, −40% and −21% (both *p* < 0.001), respectively) per main raceme compared to *2.5B*. Moreover, on average, 34% and 18% of the flowers that developed on *0.4B* on *Daktari* and *CR3153*, respectively, were deformed. The number of asymptomatic flowers on the B‐efficient *CR2267* was reduced by 12% on average on *0.4B* compared to *2.5B* (*p* = 0.119), with 8.9% of the flowers developed on *0.4B* being deformed (Figures 2 and 3A, Table S2B). Comparing the cultivars on *0.4B*, the B‐efficient *CR2267* developed more asymptomatic flowers than the B‐inefficient *Daktari* (+20%, *p* = 0.033) and *CR3153* (+10%, *p* = 0.499). *CR2267* also developed significantly fewer deformed flowers than *CR3153* (−56%, *p* < 0.001) and *Daktari* (−76%, *p* < 0.001), with *CR3153* developing fewer than *Daktari* (−46%, *p* < 0.001) at 4 DAF (Figure 3A).

Significant differences in the number of asymptomatic (*p* < 0.001) and deformed (*p* < 0.001) flowers between *0.4B* and *2.5B* persisted for all cultivars during flowering up to 14 DAF, with an increased percentage of the flowers developed on *0.4B* being macroscopically deformed (on average, *Daktari* = 61%, *CR3153* = 65%, and *CR2267* = 22%) compared to 4 DAF (Figure 3A, Table S2B). At 14 DAF, no significant differences in the number of asymptomatic flowers existed anymore on *0.4B* between the cultivars (*p* = 0.094), but *CR2267* developed 78% and 87%, respectively, significantly fewer deformed flowers on *0.4B* than *CR3153*, and *Daktari* (both *p* < 0.001) and *CR3153* developed 41% fewer deformed flowers than *Daktari* (*p* < 0.001) (Figure 3A, Table S2B).

At 4 DAF, primarily only the main racemes had started to flower, while at 14 DAF, on average, between 7 and 9 side racemes of all cultivars had developed flowers. On *0.4B*, most of the side racemes on *Daktari* and *CR3153* developed deformed flowers (92% and 64%, respectively), whereas on *CR2267*, only about 50% of the side racemes had developed deformed flowers (Figure 3B, Table S2B).

Boron concentrations of the side racemes were with < 6 mg kg^−1^ DW, on average, four times lower on *0.4B* compared to *2.5B* (*p* < 0.001) with no significant differences between the cultivars (*p* = 0.918) (Figure 3C, Table S2B). Boron concentrations of < 6 mg kg^−1^ DW are typically found in rapeseed plants with a B‐deficient inflorescence phenotype (Jiang et al. 2025; Verwaaijen et al. 2023). The main shoot length at harvest was significantly reduced for each cultivar on *0.4B* compared to *2.5B* (Figure 3D, Table S2B).

### 3.2 Boron‐Deficiency in Rapeseed Inflorescences Reduced the Abundance of Visiting Pollinators With the B‐Efficient Cultivar Being Less Affected Than the B‐Inefficient Cultivars

**FIGURE 4:**
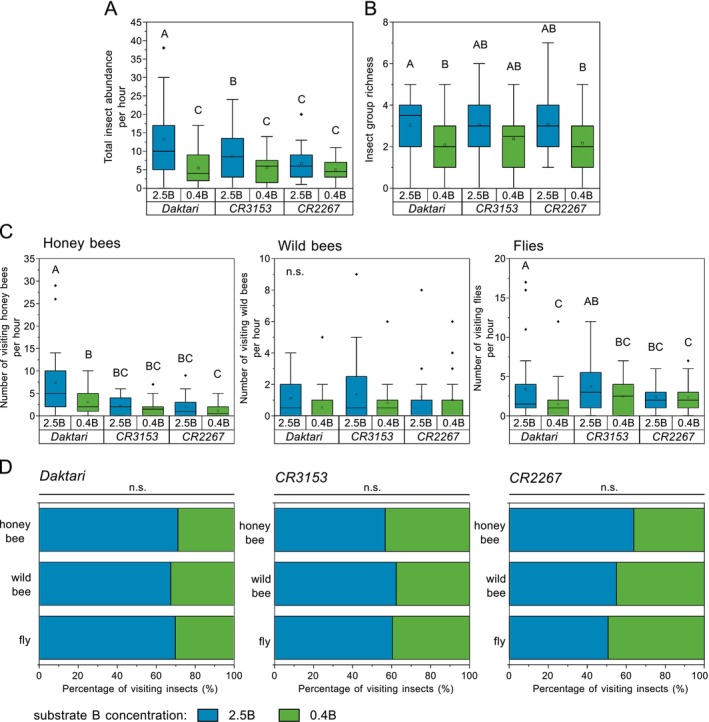
Boron (B)‐level and *Brassica napus* cultivar‐dependent pollinator observations. (A) Total insect visitor abundance per hour and (B) group richness of insects, (C) number of observed honey bees, wild bees, and flies averaged per hour detected on cultivars *Daktari*, *CR3153*, and *CR2267* at B‐sufficient (2.5 mg B kg^−1^ substrate; *2.5B*) and B‐deficient (0.4 mg B kg^−1^ substrate; *0.4B*; and for *Daktari* additionally 0.25 mg B kg^−1^ substrate; *0.25B*) levels, and (D) sum of all visiting insects of honey bees, wild bees, and flies for each cultivar *Daktari*, *CR3153*, and *CR2267* expressed as a percentage at B‐sufficient (*2.5B*) and B‐deficient (*0.4B*; and for *Daktari* additionally *0.25B*) levels. Values were determined by observing 40 individual plants per cultivar and B level for 1 h. For (A–C): Upper‐case letters indicate significant differences between cultivars and B levels (*p* < 0.05, GLMM, emmeans for pairwise post hoc comparisons, post hoc Tukey's test). For (D): Pearson's chi‐squared test between the B level and insect groups for each cultivar; n.s.: Not significant (for [A, B]: *n* = 136; [C]: *n* = 18–26; and [D]: *n* = 14–194).

In total, 1031 insect visitors were observed across cultivars and substrate B levels, with honey bees (42%), flies (35%), and wild bees (13%) most frequently observed (Table S3). Total insect visitor abundance per hour (determined for 40 plants per cultivar and B level) was higher for plants grown on *2.5B* than on *0.4B* (Figure 4A, Table S2C): for *Daktari* by +59% (*p* < 0.001), for *CR3153* by +37% (*p* = 0.002), and for *CR2267* by +27% (*p* = 0.252), on average. On *2.5B*, *Daktari* was visited by a significantly higher number of insects, with 13.2 insects per hour on average compared to 8.7 and 6.7 for *CR3153* and *CR2267*, respectively (for both, *p* < 0.001). On *0.4B*, no significant differences in insect visitor abundance were found between cultivars (*p*‐values between 0.393 and 0.991), with the number of insects observed ranging between 4.9 and 5.5 per hour (Figure 4A).

While the B level had an overall significant effect across cultivars with a lower insect group richness on *0.4B* than on *2.5B* (*p* < 0.001), insect group richness for individual cultivars was significantly reduced only for *Daktari* on *0.4B* (*p* = 0.026) (*CR3135*: *p* = 0.588; *CR2267*: *p* = 0.387). No significant differences in the insect group richness were observed between cultivars within each B level (Figure 4B, Table S2C).

Honey bee abundance on *2.5B* was, on average, significantly higher for *Daktari* (7.5 honey bees per hour) than for *CR3153* and *CR2267* (2.3 and 2.1, respectively) (for both *p* < 0.001). However, a limited B supply level of *0.4B* reduced honey bee abundance by 50%, on average, compared to *2.5B* across cultivars, which was significant for *Daktari* (reduced by 60%, *p* < 0.001), but not for *CR3135* (*p* = 0.81) and *CR2267* (*p* = 0.309) (Figure 4C, Table S2C). The number of flower‐visiting wild bees ranged between 0.5 and 1.4 per hour on average, and was not significantly affected by the B level across cultivars. However, wild bee abundance tended to be lower on *0.4B* compared to *2.5B* for *Daktari* and *CR3153* by more than 40% on average (Figure 4C, Table S2C). Fly abundance per hour was significantly reduced on *0.4B* compared to *2.5B* for *Daktari* by 57% on average (*p* < 0.001), but not for *CR3153* (*p* = 0.101) and *CR2267* (*p* = 0.983) (Figure 4C, Table S2C).

The total number of all insect visitors and also the percentage of visitors of individual insect groups (i.e., honey bees, wild bees, and flies), observed over the whole observation period, was lower on B‐deficient conditions for *Daktari* and *CR3153* compared to B‐sufficient conditions; while for *CR2267*, only the percentage of visiting honey bees was reduced (Figure 4D). However, no significant association between the B level and abundance of the respective insect group was found for any cultivar (*Daktari*: chi‐square = 0.256, df = 2, *p* = 0.88; *CR3153*: chi‐square = 0.534, df = 2, *p* = 0.766; *CR2267*: chi‐square = 2.448, df = 2, *p* = 0.294; Pearson's chi‐squared test) (Figure 4D).

### 3.3 Insect Visitor Abundance Decreased More as the Rapeseed Plants Suffered From B‐Deficiency

**FIGURE 5:**
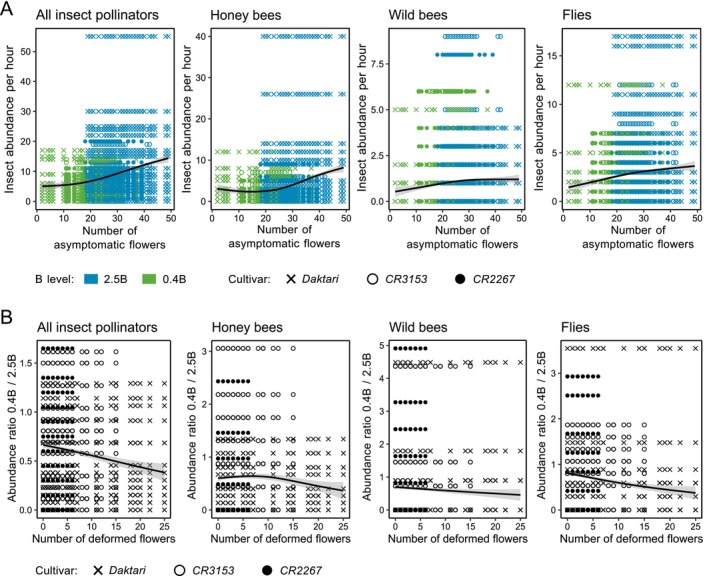
Correlation between *Brassica napus* flower phenotypes and insect abundance depending on the boron nutritional status. (A) Abundance of all insect pollinators, honey bees, wild bees, and flies per hour against the number of asymptomatic flowers (4 days after flowering; DAF) and (B) ratio *0.4B* to *2.5B* (0.4 [and for *Daktari* additionally 0.25 mg B kg^−1^ substrate] and 2.5 mg B kg^−1^ substrate) of abundance per hour of all insect pollinators, honey bees, wild bees, and flies against the number of deformed flowers (4 DAF) of rapeseed cultivars *CR2276*, *CR3153*, and *Daktari* at B‐sufficient (*2.5B*) and B‐deficient (*0.4B*; and for *Daktari* additionally 0.25 mg B kg^−1^ substrate; 0.25B) levels. Insect abundance values were determined by observing 40 plants per cultivar and B level for 1 h. (For [A]: *p* < 0.05, GLMM, *n* = 5602; and for [B]: Spearman correlation, *n* = 2186).

The numbers of asymptomatic and deformed flowers were negatively correlated (*r* = −0.56, *p* < 0.001); that is, the more macroscopic B‐deficiency symptoms, indicated by an increasing number of deformed flowers, the more the number of asymptomatic flowers decreased (Figure S1). The response of insect visitor abundance to B treatment was greatest for *Daktari*, followed by *CR3153* and *CR2267*, with more insects generally observed on *2.5B* than *0.4B* (Figure 3A). Similarly, the absolute number of asymptomatic flowers counted in these genotypes at 4 DAF was highest for *Daktari*, followed by *CR3153* and *CR2267*, with more asymptomatic flowers generally observed on *2.5B* than *0.4B* (Figure 4A). Similar B‐dependent insect visitor abundance patterns between the three cultivars were identified for the three most frequently observed pollinator groups, that is, honey bees, wild bees, and flies (Figure 5A). Honey bee visit abundance increased most with an increase in the number of asymptomatic flowers (*p* = 0.0015), followed by fly visit abundance (*p* = 0.0065), while wild bee visit abundance (*p* = 0.354) was least affected by the number of asymptomatic flowers (Figure 5A, Table S2D). The interaction between the abundances of different insect groups and the number of asymptomatic flowers was cultivar‐dependent (*p* < 0.001) (Table S2D).

Moreover, with an increasing level of B‐deficiency symptoms in the inflorescence, that is, an increasing number of deformed flowers developing on *0.4B*, the total insect visitor (*r* = −0.111, *p* < 0.001) and fly (*r* = −0.158, *p* < 0.001) abundance decreased in treatment *0.4B* compared to *2.5B*, while the honey bee (*r* = 0.0003, *p* = 0.988) and wild bee visit abundances were not affected (*r* = −0.017, *p* = 0.436) by these two B supply levels averaged across all cultivars (Figure 5B). In summary, total insect visitor abundance correlated positively with the number of asymptomatic flowers (*p* < 0.001) (Figure 5A, Table S2D) and thus depended on both (1) the B level in the substrate (as the number of asymptomatic flowers increased with an increasing B level), and (2) the cultivar (Figure 3A).

### 3.4 Insect‐ Compared to Self‐Pollination Led to Higher Seed Yield in B‐Inefficient Rapeseed Cultivars

**FIGURE 6:**
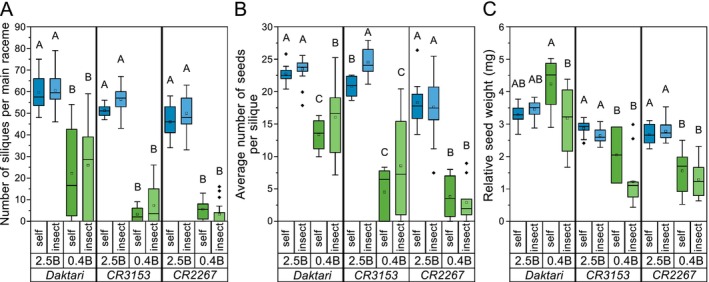
Boron (B)‐level and pollinator treatment‐dependent yield of *Brassica napus* rapeseed plants. (A) Number of siliques per main raceme, (B) average number of seeds per silique, and (C) relative seed weight for each cultivar (*Daktari*, *CR3153*, and *CR2267*) at B‐sufficient (2.5 mg B kg^−1^ substrate; *2.5B*) and B‐deficient (0.4 mg B kg^−1^ substrate; *0.4B*) levels and comparing insect pollination (insect) to self‐pollination (self). Upper‐case letters indicate significant differences between the B levels and pollination treatments for each cultivar (*p* < 0.05, LME, emmeans for pairwise post hoc comparisons, and post hoc Tukey's test) (self‐pollination *n* = 3–10, insect pollination *n* = 8–24).

The number of well‐developed siliques with seeds per main raceme was significantly higher on *2.5B* (46–60 siliques) for each cultivar than on *0.4B* with 22–25 siliques for *Daktari* and 3–7 siliques for *CR3153* and *CR2267* (all *p* < 0.001) (Figure 6A, Table S2E). The number of siliques with seeds at each B level did not significantly differ between insect‐ and self‐pollination (*2.5B*: *p* = 0.147, *0.4B*: *p* = 0.32) for each cultivar (Figure 6A).

Sufficient B supply led to a significantly higher quantity of seeds per silique (on average 21 seeds) than B‐deficiency (on average 8 seeds) for each cultivar (Figure 6B) (all *p* < 0.001, Table S2E). The seed yield per silique for *Daktari* (*p* = 0.03) and *CR3153* (*p* = 0.0496) was significantly higher with insect pollination by 20% and 18% on B‐deficient and B‐sufficient levels compared to self‐pollination, respectively (Figure 6B). For *CR2267*, no significant effect of pollination type on seed yield was found (Figure 6B, Table S2E).

The B supply level had a significant effect on the relative seed weight for *CR3153* and *CR2267* (both *p* < 0.001, Table S2E), with a 41% and 48% lower relative seed weight on *0.4B* than on *2.5B* across pollination types, respectively (Figure 6C), but not for *Daktari* (*p* = 0.942, Table S2E). Insect pollination did not impact the relative seed weight across B supply levels (Table S2E), only for a significantly decreased relative seed weight for *Daktari* on *0.4B* (*p* = 0.016) (Figure 6C).

## 4 Discussion

In this study, we used different rapeseed cultivars that have been demonstrated based on their ability to form biomass under B‐limiting soil conditions during the vegetative juvenile plant growth stage to be more (*CR2267*) or less (*Daktari* and *CR3153*) B‐deficiency tolerant and to investigate how the B nutritional status affects the reproductive performance of rapeseed plants both directly (via flower fertility) and indirectly (via changes in their interactions with insects, including pollinators). Our findings demonstrate that a reduced B availability during flowering has strong negative consequences for rapeseed plants by impairing (1) growth and plant‐intrinsic fertility, and (2) their attractiveness for beneficial pollinators, and hence, pollination and seed set.

### 4.1 Boron‐Deficiency Negatively Affects Floral Traits, Fertility, and Seed Set of Rapeseed Cultivars

Typical B‐deficiency symptoms found in rapeseed (Lou et al. 2011; Jiang et al. 2025; Verwaaijen et al. 2023; Zhang et al. 2014) were observed in this study on *0.4B*: a reduced number of asymptomatic flowers, the development of deformed flowers, and a reduced fruit and seed set, typical for the “flowering without seed setting” phenotype. The B‐efficient cultivar *CR2267* showed significantly less B‐deficiency symptoms in flower parameters compared to the B‐inefficient cultivars *CR3153* and *Daktari*. Concerning the parameter seed yield under self‐pollination conditions for the oilseed‐type cultivars in our study, *Daktari* developed, especially on *0.4B*, more siliques and seeds compared to *CR3153*. This may be explained by the fact that *Daktari* is a more modern hybrid oilseed‐type variety that was specifically bred for high yield. Compared to the oilseed‐types *CR3153* and *Daktari*, *CR2267* had a lower fruit and seed set on both B supply levels. This may be due to the fact that *CR2267* is a fodder‐type cultivar which, like other fodder or forage crops, is generally characterized by the ability to grow rapidly, form large leaves, and accumulate a higher vegetative biomass for feed purposes, but which has not been bred for high seed yield as oilseed‐types have been (Abdelrahman et al. 2022; Soengas et al. 2008; Zampaligré et al. 2021).

### 4.2 Insect Visitor Abundance and the Effect of Insect Pollination on the Yield of Rapeseed Is Cultivar Dependent

The three investigated rapeseed cultivars significantly differed in insect visitor abundance and the effect of insect pollination on their yield. On *2.5B*, the highest numbers of visiting insects were observed on *Daktari* flowers, and slightly more insects were observed on *CR3153* than on *CR2267* flowers. As flower visitor abundances were strongly and positively correlated with the number of asymptomatic, healthy appearing flowers across cultivars, the higher number of asymptomatic flowers might explain the higher insect visitor abundance for *Daktari* compared to *CR3153* and *CR2267* on *2.5B*. Honey bees (41.51%), flies (34.92%), and wild bees (13.19%; excluding bumblebees) were the main groups of pollinators visiting the rapeseed plants, followed by bumblebees with 4.17% of all visiting insects. Honey bees and other Apidae taxa, such as bumblebees, mining bees, and sweat bees, are known to be the main visitors of rapeseed plants (Badenes‐Pérez 2022; Garratt et al. 2014; Kazda et al. 2019), likely because of their rich nectar production known to attract them (Osterman et al. 2021). Hoverflies are also known to frequently visit rapeseed flowers (Bommarco et al. 2012; Garratt et al. 2014; Langridge and Goodman 1982), but they only made up 3.6% of all visitors observed in our study.

Several studies observed an increase in seed yield per rapeseed plant with cross‐pollination, mainly via insect pollination, compared to self‐pollination (Badenes‐Pérez 2022; Bommarco et al. 2012; Fairhurst et al. 2022; Garratt et al. 2014, 2018; Hudewenz et al. 2013). In our study, seed set increased with insect pollination compared to self‐pollination for *Daktari* and *CR3153* (both oilseed‐type rapeseeds) on *0.4B* and *2.5B*, respectively. However, no significant effect of insect versus self‐pollination on seed set was found for the fodder‐type variety *CR2267*. The seed set was lower in *CR2267* than for the other cultivars, although the numbers of asymptomatic flowers and insect visitation on *2.5B* were similar to *CR3153*, indicating that this cultivar generally has lower flower fertility, which cannot be compensated for by insect pollination. This finding agrees with other studies showing that the effect of insect pollination on yield may differ between rapeseed cultivars (Hudewenz et al. 2013; Langridge and Goodman 1982). While such cultivar‐dependent effects of insect pollination are often ignored in crop cultivation (Klein et al. 2007; Ouvrard and Jacquemart 2019), our results and those of others clearly demonstrate the need to consider such yield dependencies of cultivars on pollinators, particularly when cultivating rapeseed in an environment with pollinator decline (Turo et al. 2024). Additionally, as hybrid cultivars are becoming the predominant rapeseed cultivars globally, the question of differences in pollinator dependencies between hybrid and open‐pollinated cultivars arises, namely whether pollinators visit hybrids less than open‐pollinated cultivars (Adamidis et al. 2019; Kazda et al. 2019). In our results, differences in pollinator dependency are rather cultivar‐dependent than dependent on the type of the breeder's seed production, as insect pollination increased the seed yield of *Daktari*, a hybrid cultivar, and *CR3153*, an open‐pollinated cultivar, while the open‐pollinated cultivar *CR2267* did not seem to be affected by insect pollination. Studies specifically investigating differences in pollinator dependency between hybrid and open‐pollinated varieties are in line with the results observed in our study (Adamidis et al. 2019; Hudewenz et al. 2013; Kazda et al. 2019). Differences in insect pollination dependencies on the seed set efficiency between cultivars might be better explained by the differences in their floral biology than by the type of the breeder's seed production. For example, the two self‐compatible rapeseed hybrids, *Hyola 61* and *Hyola 420*, differ in their durations of anthesis, with the latter displaying a 3× shorter anthesis period (Blochtein et al. 2014). Compared to the self‐pollination treatment, the increase in productivity with insect pollination was twice as high for *Hyola 61* than for *Hyola 420*, probably due to the longer anthesis period of the former (Blochtein et al. 2014). Consequently, it was hypothesized that *Hyola 61* is more dependent on insect pollination than *Hyola 420* due to a different floral biology. Further research on the cultivars in our study is necessary to evaluate whether differences in floral biology cause the different dependencies on insect pollination.

Relative seed weight was partly higher for self‐pollinated flowers and thus negatively associated with seed set for *Daktari* and *CR3153*. This suggests that plants that had fewer seeds due to the lack of beneficial insect pollination were able to allocate the available resources to these fewer seeds, leading to the formation of heavier seeds compared to insect‐pollinated plants with more but consequently smaller and lighter seeds (Badenes‐Pérez 2022; Bommarco et al. 2012; Hudewenz et al. 2013).

### 4.3 Boron‐Deficiency Negatively Affects Insect Visitor Abundance in Rapeseed

Boron is crucial for flower development (Dell and Huang 1997; Zhang et al. 2017). In our study, B‐deficient conditions led to the development of deformed flowers concomitant with a reduction in the number of asymptomatic flowers and with a direct negative impact on insect visitor abundance, as flower visitor abundances were strongly and positively correlated with the number of asymptomatic flowers across cultivars. The sum of all visitations, either by all main insect groups together or individually for honey bees and flies, was positively correlated with the number of asymptomatic flowers and was thus higher on *2.5B* compared to *0.4B*. This was especially true for *Daktari* and *CR3153*, which exhibited a strongly decreased number of asymptomatic flowers compared to *CR2267* on *0.4B*. In contrast, the abundance of visiting wild bees was less strongly positively correlated with the numbers of asymptomatic flowers, which may suggest that they are attracted by floral signals other than only the appearance of asymptomatic flowers. The development of deformed flowers might additionally explain the reduction in fly or wild bee abundance from *2.5B* to *0.4B*, as especially fly abundance was negatively correlated with the number of deformed flowers. This highlights the importance of well‐developed healthy flowers for attracting pollinators and ensuring cross‐pollination by insects, as the development of deformed flowers under B‐deficiency seems to decrease attractiveness.

To date, most research on the effect of mineral nutrition and insect pollination focused on synergistic effects between macronutrient (e.g., N, P, and K) supplementation and insect pollination in rapeseed (Burkle and Irwin 2010; Dupont et al. 2018; Garratt et al. 2018; Russo et al. 2023; Sengupta and Krishna 2023), for example, reduced plant NPK fertilization negatively impacts the amount of pollen and nectar (Russo et al. 2023). However, little is known about the effect of reduced plant B supply on plant–insect interactions. For instance, the application of B was found to increase the volume and sugar concentration of nectar in red clover compared to plants grown on B‐deficient soil conditions (Eriksson 1979; Stoltz and Wallenhammar 2013) and thus positively affected the flowers' attractiveness to pollinators. Similarly, in a field study, drought stress, known to induce B‐deficiency, was shown to negatively affect plant–pollinator interactions in common wildflowers by reducing flower‐reward production (Descamps et al. 2018; Gallagher and Campbell 2017; Phillips et al. 2018). Our findings provide additional insight into the hitherto little‐known role of B nutritional conditions on plant–insect interactions.

Besides a negative impact of B‐deficiency on floral rewards, floral cues can also be impaired by B‐deficiency. Rapeseed flowers emit a scent, which is very attractive, especially to bees (Cook et al. 2005; Mussury and Fernandes 2000). Since floral scent composition in rapeseed is altered, for example, by increasing N and water supply (Höfer et al. 2022), a similar floral scent alteration upon B‐deficiency may be explained by changes in rapeseed pollen, which are known to emit odor to attract insects (Cook et al. 2005), besides their primary function as a source of protein, lipids, vitamins, and minerals (Abrol 2007). Boron‐deficiency can lead to the development of hollow pollen grains (Dell and Huang 1997), which may not be attractive to pollinators, thus potentially explaining the reduced insect visits on B‐limited plants in our study. Additionally, B‐deficiency affected the flower shape and appearance, leading to, for example, wrinkled petals. Similarly, sulfur deficiency in rapeseed and *Brassica rapa* is known to affect flower shape with a reduced flower size and, furthermore, to reduce pigmentation of the typically vibrantly yellow‐colored flowers, which are then likely perceived as less colorful, and therefore, less attractive to pollinators (Ausma et al. 2021; Haneklaus et al. 2005).

The importance of floral signals may explain why changes in insect visitor abundance between *2.5B* and *0.4B* have been cultivar and B‐efficiency‐dependent. For example, flower visitor abundance decreased more strongly under B‐limitation for the B‐inefficient *Daktari* than for the B‐efficient *CR2267*, the latter of which displayed fewer B‐deficiency symptoms. Future research will be needed to disentangle cultivar‐ and B‐dependent effects of flower size, shape, color, nectar, and pollen content, as well as scent, on flower attractiveness to pollinators.

Boron is also crucial for successful seed set as B‐deficiency may prevent ovule fertilization even after successful pollination, for example, due to an arrested pollen tube growth (Dell and Huang 1997; Lou et al. 2011). It is assumed that insect pollination leads to a higher proportion of pollen reaching the stigma than upon self‐pollination (Pechan 1988; Zou et al. 2017). Accordingly, an increased seed set on both *2.5B* and *0.4B* was observed for *Daktari* and *CR3153*. In agreement with our results, the application of B to strawberries and olives led to a higher pollen viability and, in combination with insect pollination, to a higher yield (Sarıdaş et al. 2021; Spinardi and Bassi 2012). However, while insect pollination may increase the seed yield of optimally fertilized plants, it does not necessarily compensate for an inadequate B nutritional status of inflorescences.

## 5 Conclusion

Especially in B‐inefficient oilseed‐type rapeseed cultivars, sufficient B fertilization increased, on the one hand, the attractiveness of plants for pollinators by enabling the development of more asymptomatic flowers, which ensured sufficient pollination and, on the other hand, guaranteed B‐dependent flower‐intrinsic fertility processes needed for seed development and yield formation. The identification of genes controlling floral B‐efficiency will help to breed for pollinator attractive high and stable yielding oilseed‐type cultivars, which are less dependent on precisely timed and energy‐intensive applications of agrochemicals. Nutrient‐efficient plants will be more adapted to future climate challenges, such as more intense droughts, which are negatively affecting nutrient uptake and hence the plants' development and attractiveness to mutualists, such as pollinators (Van der Niet et al. 2023). Our results further demonstrated that the interplay between B nutrition, B‐efficiency, flower development, attractiveness to pollinators, and ultimately pollination and yield is complex, highly genotype dependent, and needs to be considered in B‐deficiency mitigation strategies.

## Author Contributions

**Jiline B. Tölle:** data curation (lead), formal analysis (lead), investigation (lead), methodology (lead), validation (lead), visualization (lead), writing – original draft (lead), writing – review and editing (equal). **Paula Prucker:** data curation (equal), formal analysis (equal), investigation (equal), methodology (equal), validation (equal), visualization (equal), writing – review and editing (equal). **Johanna Saumweber:** data curation (equal), formal analysis (equal), investigation (equal), methodology (equal), validation (equal), writing – review and editing (equal). **Thomas D. Alcock:** data curation (equal), formal analysis (equal), investigation (equal), methodology (equal), validation (equal), writing – review and editing (equal). **Sara D. Leonhardt:** conceptualization (equal), data curation (equal), formal analysis (equal), funding acquisition (equal), investigation (supporting), methodology (supporting), project administration (lead), resources (equal), supervision (lead), validation (equal), visualization (equal), writing – review and editing (lead). **Gerd Patrick Bienert:** conceptualization (lead), data curation (equal), formal analysis (equal), funding acquisition (lead), investigation (supporting), methodology (equal), project administration (lead), resources (equal), supervision (lead), validation (equal), visualization (equal), writing – review and editing (lead).

## Funding

This project was financially supported by the Bavarian State Ministry of the Environment and Consumer Protection within the project network BayKlimaFit 2 (Project 5, TEW01CO2P‐77745, and Project 8, TEW01CO2P‐77748).

## Conflicts of Interest

The authors declare no conflicts of interest.

## Supporting information


**Data S1:** ece372895‐sup‐0001‐supinfo.pdf.

**Data S2:** ece372895‐sup‐0002‐supinfo.xlsx.

## Data Availability

All data are accessible via the EXCEL file that is published alongside the article.
