# Supplementary material for: Bee on Boron—Sufficient Boron Supply of Brassica napus Is Crucial for Attracting Pollinating Insects to Ensure Seed Yield
Source: Ecol Evol. 2026 Jan 12;16(1):e72895. doi: 10.1002/ece3.72895 (PMC12793893; doi:10.1002/ece3.72895)
Supplement: Supplementary file 1 — Data S1: ece372895‐sup‐0001‐supinfo.pdf. [file ECE3-16-e72895-s001.pdf]

SUPPORTING INFORMATION

FIGURES AND TABLES

FIGURE S1

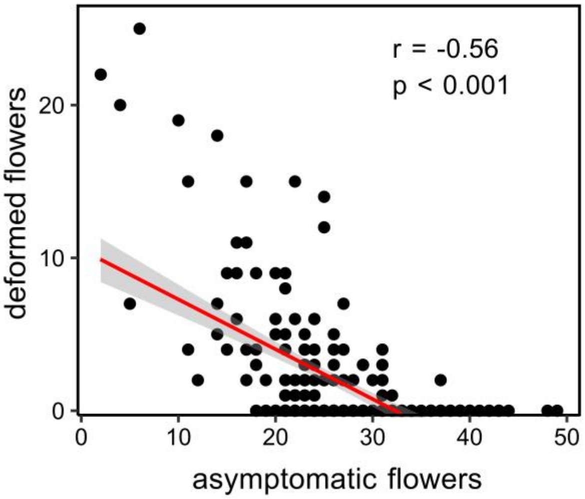

Figure S1. Spearman's correlation of the number of deformed *Brassica napus* flowers against asymptomatic flowers (per main raceme of single plant) at 4 days after flowering started across all cultivars (*Daktari*, *CR3153* and *CR2267*) and boron (B) levels (B-sufficient: 2.5 mg B kg^{-1} substrate; 2.5B; B-deficient: 0.4 mg B kg^{-1} substrate; 0.4B) (n = 251) (r correlation coefficient, p-value).

Table S1. Climate data. **(A)** Day time (> 0.0 klx light intensity) glasshouse temperature and outside temperature for the months April and May 2023. **(B)** Day time (> 0.0 klx light intensity) glasshouse temperature for the period November till March 2023 and night time (0.0 klx light intensity) glasshouse temperature for the period November till May 2023.

A

**Month**	**Glasshouse temperatur [°C]**			**Outside Temperature [°C]**		
Day (> 0.0 klx)	Mean	SD	Max	Mean	SD	Max
April	17.4	± 1.7	25.7	10.8	± 5.0	27.8
May	19.9	± 3.8	31.6	17.6	± 6.1	35.7

B

**Glasshouse temperature**		
Time	Month	[°C]
Day (> 0.0 klx)	November - March	≈ 18
Night (0.0 klx)	November - May	≈ 16

Table S2. Statistical analysis. (**A**) Summary of the Two-Way-ANOVA results of the three *Brassica napus* cultivars *Daktari*, *CR3153* and *CR2267* and boron (B) levels (B-sufficient and B-deficient) on shoot dry weight (28 days after sowing; DAS) and L3 leaf length (degrees of freedom (Df), F-value and p-value); (**B**) Summary of the Two-Way-ANOVA results of the cultivars *Daktari*, *CR3153* and *CR2267* and B levels (B-sufficient and B-deficient) on number of asymptomatic and deformed flowers of the main raceme at 4 and 14 days after flowering started (DAF), number of side racemes (SR) with asymptomatic and deformed flowers at 14 DAF, tissue B concentration and main shoot length at harvest (degrees of freedom (Df), F-value and p-value); (**C**) Summary of the generalized linear mixed model (GLMM) results for additive and interactive effects of the cultivars *Daktari*, *CR3153* and *CR2267* and B levels (B-sufficient and B-deficient) on overall insect abundance, insect group richness, and number of honey bees, wild bees and flies, tested with ANOVA (Chi-Square value, degrees of freedom (Df) and p-value); (**D**) Summary of the generalized linear mixed model (GLMM) results for additive and interactive effects of number of asymptomatic flowers, the cultivars *Daktari*, *CR3153* and *CR2267* and B levels (B-sufficient and B-deficient) on overall insect abundance, and number of honey bees, wild bees and flies, tested with ANOVA (Chi-Square value, degrees of freedom (Df) and p-value); (**E**) Summary of the linear mixed model (LMM) results for additive and interactive effects of B levels (B-sufficient and B-deficient) and pollination treatment (self and insect) on number of siliques, average number of seeds per silique and relative seed weight for each cultivar (*Daktari*, *CR3153* and *CR2267*), tested with ANOVA (degrees of freedom (Df), F-value and p-value).

A

	**Shoot dry weight**			**L3 length**		
	**Df**	**F-value**	**p-value**	**Df**	**F-value**	**p-value**
**Cultivar**	2	10.2	<0.001	2	16.8	<0.001
**B level**	1	194.1	<0.001	1	197.1	<0.001
**Interaction**	2	4.9	0.01	2	15.3	<0.001

B

	Asymptomatic flowers 4 DAF			Deformed flowers 4 DAF			Asymptomatic flowers 14 DAF			Deformed flowers 14 DAF		
	Df	F-value	p-value	Df	F-value	p-value	Df	F-value	p-value	Df	F-value	p-value
**Cultivar**	2	0.9	0.411	2	28.3	<0.001	2	2.4	0.094	2	23.8	<0.001
**B level**	1	85.2	<0.001	1	214.1	<0.001	1	67.0	<0.001	1	128.4	<0.001
**Interaction**	2	11.8	<0.001	2	28.3	<0.001	2	1.3	0.265	2	23.8	<0.001

	SR with asymptomatic flowers			SR with deformed flowers			Tissue B concentration			Main shoot length		
	Df	F-value	p-value	Df	F-value	p-value	Df	F-value	p-value	Df	F-value	p-value
**Cultivar**	2	51.6	<0.001	2	13.5	<0.001	2	0.1	0.919	2	19.5	<0.001
**B level**	1	640.0	<0.001	1	619.3	<0.001	1	90.2	<0.001	1	78.9	<0.001
**Interaction**	2	5.4	0.006	2	13.5	<0.001	2	1.0	0.403	2	1.8	0.173

C

	Insect abundance			Insect group richness		
	Chisq	Df	p-value	Chisq	Df	p-value
**Cultivar**	44.4	2	<0.001	1.2	2	0.554
**B level**	77.7	1	<0.001	15.1	1	<0.001
**Interaction**	12.0	2	0.003	1.1	2	0.591

	Honey bees			Wild bees			Flies		
	Chisq	Df	p-value	Chisq	Df	p-value	Chisq	Df	p-value
**Cultivar**	45.7	2	<0.001	1.8	2	0.413	8.0	2	0.018
**B level**	31.0	1	<0.001	3.1	1	0.079	14.1	1	<0.001
**Interaction**	3.8	2	0.152	1.5	2	0.467	6.7	2	0.035

D

	Insect abundance			Honey bees			Wild bees			Flies		
	Chisq	Df	p-value	Chisq	Df	p-value	Chisq	Df	p-value	Chisq	Df	p-value
**Asymptomatic flowers**	12.2	1	<0.001	10.1	1	0.0015	0.9	1	0.354	7.4	1	0.0065
**Cultivar**	444.9	2	<0.001	1133.4	2	<0.001	139.3	2	<0.001	47.5	2	<0.001
**B level**	206.4	1	<0.001	31.7	1	<0.001	55.2	1	<0.001	308.0	1	<0.001
**Interaction (asympt:cultivar)**	22.9	2	<0.001	28.2	2	<0.001	1.8	2	0.407	15.5	2	<0.001

E

	Number of siliques								
	Daktari			***CR3153***			***CR2267***		
	Df	F-value	p-value	Df	F-value	p-value	Df	F-value	p-value
**(Intercept)**	1	286.7	<0.001	1	744.8	<0.001	1	1009.1	<0.001
**B level**	1	32.2	<0.001	1	458.6	<0.001	1	794.7	<0.001
**Pollination**	1	0.5	0.478	1	7.7	0.009	1	0.2	0.653
**Interaction (B level:pollination)**	1	0.2	0.629	1	0.1	0.724	1	3.5	0.069

Average number of seeds per silique

	Daktari			*CR3153*			*CR2267*		
	**Df**	**F-value**	**p-value**	**Df**	**F-value**	**p-value**	**Df**	**F-value**	**p-value**
**(Intercept)**	1	842.9	<0.001	1	282.1	<0.001	1	310.3	<0.001
**B level**	1	34.6	<0.001	1	78.4	<0.001	1	117.5	<0.001
**Pollination**	1	5.2	0.030	1	12.3	0.002	1	0.7	0.408
**Interaction (B level:pollination)**	1	4.1	0.053	1	0.0	0.884	1	0.1	0.733

Relative seed weight

	Daktari			*CR3153*			*CR2267*		
	**Df**	**F-value**	**p-value**	**Df**	**F-value**	**p-value**	**Df**	**F-value**	**p-value**
**(Intercept)**	1	1112.6	<0.001	1	474.5	<0.001	1	1256.8	<0.001
**B level**	1	0.0	0.942	1	60.6	<0.001	1	93.1	<0.001
**Pollination**	1	1.5	0.231	1	4.1	0.055	1	0.0	0.851
**Interaction (B level:pollination)**	1	9.3	0.005	1	0.4	0.547	1	1.7	0.208

Table S3. List of insect count per insect group per *Brassica napus* cultivar and substrate boron (B) concentration. Insect counts are summed up per insect group for the cultivars *Daktari*, *CR3153* and *CR2267* and substrate B levels (B-sufficient: 2.5 mg B kg^{-1} substrate; 2.5B; B-deficient: 0.4 mg B kg^{-1} substrate; 0.4B, and for *Daktari* additionally 0.25 mg B kg^{-1} substrate; 0.25B) for the whole observation period (*Daktari* for 9 observation days, *CR3153* for 8 observation days, *CR2267* for 6 observation days). Total sums per insect group and per cultivar and B level. Percentage of absolute number of members per insect group of total number of insects caught (1031 = 100%) was calculated. Beetles (all Coleoptera), bumblebees (all *Bombus* spec.), butterflies (all Lepidoptera), flies (all Diptera excluding Syrphidae), honey bees (*Apis mellifera*), hoverflies (all Syrphidae) and wild bees (all Apidae excluding *Apis mellifera* and *Bombus* species). Insects (n = 36) assigned to the group "other insects" were not included in the calculation.

		Insect group							
**Cultivar**	**B level**	**Honey bee**	**Fly**	**Wild bee**	**Bumblebee**	**Hoverfly**	**Beetle**	**Butterfly**	**Sum per cultivar and B level**
Daktari	**2.5B**	194	88	29	11	9	11	0	342
	**0.4B**	79	38	14	4	4	2	0	141
CR3153	**2.5B**	55	90	33	14	9	7	0	208
	**0.4B**	42	59	20	6	4	1	0	132
CR2267	**2.5B**	37	43	22	5	8	4	1	120
	**0.4B**	21	42	18	3	3	1	0	88
**Sum per insect group (abs.)**		428	360	136	43	37	26	1	1031
	**in %**	41.51	34.92	13.19	4.17	3.59	2.52	0.10	100
